# Low-Level Human Equivalent Gestational Lead Exposure Produces Sex-Specific Motor and Coordination Abnormalities and Late-Onset Obesity in Year-Old Mice

**DOI:** 10.1289/ehp.10862

**Published:** 2007-12-07

**Authors:** J. Leigh Leasure, Anand Giddabasappa, Shawntay Chaney, Jerry E. Johnson, Konstantinos Pothakos, Yuen Sum Lau, Donald A. Fox

**Affiliations:** 1 Department of Psychology; 2 Department of Biology and Biochemistry and; 3 College of Optometry, University of Houston, Houston, Texas, USA; 4 Department of Natural Sciences, University of Houston-Downtown, Houston, Texas, USA; 5 Department of Pharmacological and Pharmaceutical Sciences, University of Houston, Houston, Texas, USA

**Keywords:** aging, amphetamine, balance, dopamine, fetal, sex, gestation, lead, motor activity, obesity

## Abstract

**Background:**

Low-level developmental lead exposure is linked to cognitive and neurological disorders in children. However, the long-term effects of gestational lead exposure (GLE) have received little attention.

**Objectives:**

Our goals were to establish a murine model of human equivalent GLE and to determine dose–response effects on body weight, motor functions, and dopamine neurochemistry in year-old offspring.

**Methods:**

We exposed female C57BL/6 mice to water containing 0, 27 (low), 55 (moderate), or 109 ppm (high) of lead from 2 weeks prior to mating, throughout gestation, and until postnatal day 10 (PN10). Maternal and litter measures, blood lead concentrations ([BPb]), and body weights were obtained throughout the experiment. Locomotor behavior in the absence and presence of amphetamine, running wheel activity, rotarod test, and dopamine utilization were examined in year-old mice.

**Results:**

Peak [BPb] were < 1, ≤ 10, 24–27, and 33–42 μg/dL in control, low-, moderate- and high-dose GLE groups at PN0–10, respectively. Year-old male but not female GLE mice exhibited late-onset obesity. Similarly, we observed male-specific decreased spontaneous motor activity, increased amphetamine-induced motor activity, and decreased rotarod performance in year-old GLE mice. Levels of dopamine and its major metabolite were altered in year-old male mice, although only forebrain utilization increased. GLE-induced alterations were consistently larger in low-dose GLE mice.

**Conclusions:**

Our novel results show that GLE produced permanent male-specific deficits. The nonmonotonic dose-dependent responses showed that low-level GLE produced the most adverse effects. These data reinforce the idea that lifetime measures of dose–response toxicant exposure should be a component of the neurotoxic risk assessment process.

Removal of lead from gasoline and other environmental sources has decreased the median blood lead concentration ([BPb]) of children in the United States < 10 μg/dL: the current low level of concern [Centers for Disease Control and Prevention (CDC) 1991]. However, there is compelling cross-sectional and prospective epidemiological evidence that [BPb] in children ≤ 10 μg/dL causes cognitive decline (Canfield et al. 2003; Hu et al. 2006; Lanphear et al. 2005; Rothenberg et al. 2002; Winneke et al. 1990). Recently, Gilbert and Weiss (2006) suggested that the BPb action level in children should be 2 μg/dL. Developmental lead exposure also has been linked to a variety of neurological and neurodegenerative disorders in children and adolescents, including attention deficit hyperactivity disorder (ADHD) (Braun et al. 2006), auditory and language impairments (Dietrich et al. 1992; Rothenberg et al. 2000; Yuan et al. 2006), retinal deficits (Rothenberg et al. 2002), neuromotor dysfunction (Bhattacharya et al. 1990, 2006; Ris et al. 2004), and schizophrenia (Opler et al. 2004). Few studies, however, have examined the long-term effects of low-level gestational lead exposure (GLE) despite findings that children with prenatal lead exposure have reduced cognitive functions (Baghurst et al. 1992; Hu et al. 2006; Schnaas et al. 2006; Wasserman et al. 2000), neuromotor and visual motor dysfunction (Ris et al. 2004; Wasserman et al. 2000), and altered auditory and retinal function (Dietrich et al. 1992; Rothenberg et al. 2000, 2002; Wasserman et al. 2000).

Maternal lead exposure results from inhalation, diet, and/or eating in lead-contaminated work areas (Correa et al. 2006; Min et al. 1996). Maternal skeletal bone lead from prior exposure mobilizes during pregnancy and lactation (Manton et al. 2003). Lead easily crosses the placental and mammary barriers (Bornschein et al. 1977; Korpela et al. 1986). Thus, the developing fetus and child are at risk, as evidenced by findings that fetal and maternal [BPb] are similar (Korpela et al. 1986).

The adverse cognitive consequences of prenatal and postnatal exposure to moderate-level ([BPb] 11–39 μg/dL) and high-level ([BPb] ≥ 40 μg/dL) lead have been studied in rodents (Cory-Slechta 1997; Crofton et al. 1980; Kuhlmann et al. 1997; Wasserman et al. 2000). Several reports also link moderate-to-high-level lead exposure to altered motor activity (Crofton et al. 1980; Ma et al. 1999) and dopaminergic signaling (Antonio and Leret 2000; Cory-Slechta 1997). To date, there are no experimental studies on the effects and mechanisms of low-level GLE on neuromotor function, despite evidence that low-level lead exposure produces these deficits in children (*vide supra*).

In this report we present a new model of human equivalent GLE and the sex-specific physiological, behavioral, and neurochemical abnormalities in year-old GLE mice. These studies were conducted because the long-term consequences of GLE are unknown and increasing evidence indicates that early developmental exposure to neurotoxicants accelerates age-related functional decline and/or produces delayed neurotoxicity (Barone et al. 1995; Basha et al. 2005; Landrigan et al. 2005; Newland and Rasmussen 2000; Rice and Barone 2000; Weiss 1990; Weiss et al. 2002). Sex differences were examined because *a*) early developmental lead exposure produces a heightened risk for attention, visual motor, and fine-motor deficits in males (Bhattacharya et al. 1990, 2006; Ris et al. 2004); *b*) male and female animals exhibit differences in exposure and susceptibility to chemicals and lead neurotoxicity (Cory-Slechta et al. 2004; Vahter et al. 2007); and *c*) this is an important and underexplored area of toxicology (Vahter et al. 2007). Our results show that GLE produced age-related, sex-specific, and non-monotonic dose–response alterations.

## Materials and Methods

### Animals

All experimental and animal care procedures were in compliance with the National Institutes of Health (NIH) *Public Health Service Policy on Humane Care and Use of Laboratory Animals* (NIH 2002) and approved by the Institutional Animal Care and Use Committee of the University of Houston. All animals were treated humanely and with regard for alleviation of suffering. Five-week-old female and male C57BL/6 mice (Harlan Sprague Dawley, Inc., Indianapolis, IN) were housed in a room with a 12:12-hr light:dark cycle as described previously (He et al. 2003). Two animal models were used: GLE and post-natal-only lead exposure (PLE). For each, dams were mated with a single male overnight, and the presence of a vaginal plug was recorded as gestational day 0.5. Dams were weighed twice weekly until postnatal day (PN) 21 (weaning). On the day of birth (PN0), the number of pups and the sex and weight of the offspring were recorded (12–18 litters per group). On PN1, litters were culled to six pups with equal number of males and females when possible. Pups were weighed on PN7, 10, and 21. At weaning, male and female mice were independently housed four to five per cage and weighed at 2, 6, 10, and 12 months of age. Control and lead drinking bottles were weighed and replaced every other day.

### Gestational lead exposure model

**Figure 1:**
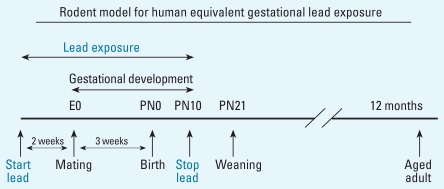
Gestational lead exposure (GLE) paradigm. Female mice were exposed to lead for 14 days prior to conception to establish a steady-state blood lead level before mating. After mating, dams were exposed to lead throughout gestation, and embryonic day 0 (E0) exposure was continued from birth (PN0) until PN10. This GLE model ensures that offspring were exposed for a period equivalent to the duration of human gestation.

Two weeks after arrival, female mice were singly housed and randomly divided into four experimental groups: one control and three GLE groups. Control dams received tap water, and GLE dams received one of three lead acetate drinking solutions (Fisher Scientific, Pittsburgh, PA): 0.005% (27 ppm lead = low-dose GLE), 0.01% (55 ppm lead = moderate-dose GLE), or 0.02% (109 ppm lead = high-dose GLE). Lead drinking solutions were provided to dams 2 weeks prior to mating to ensure [BPb] stabilization and a lead body burden throughout gestation and until PN10. Control males were used once for breeding with lead-exposed dams. We selected the pre-natal through PN10 period for our GLE study (Figure 1) because rodent brain and retinal development during this period is equivalent to that during human gestation (Dobbing and Sands 1979; Raedler and Sievers 1975; Rice and Barone 2000). Our overall goal was to compare the behavioral and neurochemical changes in low-level and high-level year-old GLE mice with those in controls. Therefore, these sets of studies were not conducted in the moderate-level GLE group.

### Postnatal lead exposure model

This model was established to compare the [BPb] profiles and body weight measures with those in GLE mice. Four weeks after arrival, female mice were singly housed and randomly divided into three experimental groups: one control and two PLE groups. Upon delivery and throughout lactation (PN0–21), PLE dams received either a 0.005% (low-dose PLE) or 0.01% (moderate-dose PLE) lead drinking solution.

### Blood lead concentrations

After decapitation, we measured trunk [BPb] in GLE dams after 14 days of lead pretreatment and on PN0. Trunk [BPb] was measured in GLE offspring at PN0, 10, 21, 30, 60, and 1 year of age and in PLE offspring at PN7, 14, 21, 30, 60, and 1 year of age. Values (micrograms per deciliter) represent the mean ± SE for 10–15 male and female mice per age per group. Samples were analyzed by anodic stripping voltammetry using LeadCare Kit I (sensitivity, ≤ 1 μg/dL; Environmental Sciences Associates, Inc., Chelmsford, MA).

### Exploratory activity: baseline and amphetamine induced

Exploratory activity was assessed in year-old male and female mice (six to nine per sex per group). Activity was measured in a fully enclosed Optovarimax behavioral monitor (40 × 40 × 40 cm; 16 infrared photo-receptor beams per X–Y side; Columbus Instruments, Columbus, OH) located in a quiet room with dim lighting. Data were recorded based on the number of laser-beam breaks made each 5-min period. Novel exploratory activity data were collected for 30 min after a 15-min acclimation period. Two weeks later, the same animals were given a 15-min acclimation period, weighed, and injected (ip) with 3 mg/kg *d*-amphetamine sulfate (Sigma A-5880; Sigma-Aldrich, St. Louis, MO), and placed in the recording chamber. Five minutes later, a 3-hr locomotor activity recording began. This dose of amphetamine avoided stereotypic behavior from competing and interfering with locomotor behavior (Zhu et al. 2006).

### Rotarod

Interlimb balance and coordination were assessed in year-old male and female mice (six to eight per sex per group) with a rotarod (Columbus Instruments). Mice were trained to stay on top of a stationary rod (3-cm diameter), then at a constant speed of 5 rpm for 90 sec. Mice were tested 3 times the day after they mastered the task, with a 1-hr intertrial interval. For each trial, the rod rotated at 5 rpm for 30 sec, and the speed was increased 0.1 rpm until the mouse fell off. The three trials were averaged to obtain one rotarod latency score (seconds) for each mouse.

### Running wheel activity

Year-old male mice (six per group) were given access to Wahman-type running wheels equipped with counters that recorded the distance (meters) traveled. Running wheels were in a room designated solely for this purpose. For five consecutive dark cycles, individual mice were placed in clean cages with an attached exercise wheel and food and water.

### High-performance liquid chromatography (HPLC) studies

Four weeks after the last behavioral experiment, aged male mice (four to seven per treatment group) were sacrificed by decapitation between 1000 hours and 1200 hours to avoid possible circadian effects. Brains were rapidly removed, and striatum and forebrain samples were dissected and placed in ice-cold 0.2 N perchloric acid and frozen at −80°C. Frozen samples were homogenized and centrifuged at 4°C. The supernatant was analyzed for the concentration of dopamine ([DA])and its major metabolite 3,4-dihydroxyphenylacetic acid ([DOPAC]) by HPLC with electrochemical detection as described (Petroske et al. 2001). DA and DOPAC are expressed as nanograms per milligram protein. [DOPAC]/[DA] ratios were used as a measure of DA utilization (Boireau et al. 1990).

### Statistical analysis

Body weight, behavioral, and neurochemical studies used one animal per litter, and the group data were analyzed by a one-way analysis of variance (ANOVA), with or without repeated measures. Male and female [BPb] data were analyzed using a two-way ANOVA. After ANOVA analyses, post-hoc multiple comparisons used the Tukey Honestly Significant Difference test (KaleidaGraph; Synergy Software, Reading, PA; Minitab Inc., Prentice-Hall, Edgewood Cliffs, NJ). Data are presented as mean ± SE, and the difference from controls was considered significant at *p* < 0.05.

## Results

### Animal models and blood lead levels

Fluid consumption and body weight of GLE dams exposed to water or lead from 14 days prior to conception until birth were measured, and gestational and litter measures were recorded. All control values (Table 1) were similar to those previously reported (Kelley and Middaugh 1996; Middaugh et al. 1988). There were no statistical differences between control and GLE groups on any measure (data not shown).

**Figure 2:**
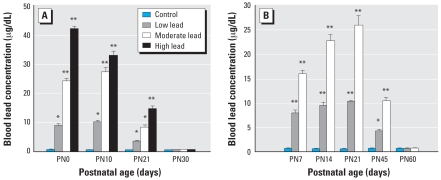
Blood lead concentration of GLE (*A*) and PLE (*B*) offspring. (*A*) GLE groups at PN0 and 10 but not at PN30 were significantly different from controls. (*B*) PLE groups at PN7, 14, 21, and 45 but not PN60 were significantly different from controls. For this and all subsequent figures, lead acetate drinking solutions contained the following: low lead = 0.005% (27 ppm), moderate lead = 0.01% (55 ppm), and high lead = 0.02% (109 ppm). Values are mean ± SE. *p < 0.05; **p < 0.01.

A two-way ANOVA revealed that [BPb] of male and female littermates were not significantly different for any GLE or PLE treatment condition. Figure 2A shows that control, and low-, moderate-, and high-dose GLE produced concentration-dependent increases in [BPb] at PN0–10, with peak [BPb] of ≤ 1, ≤ 10, 27, and 42 μg/dL, respectively. By PN30, [BPb] in GLE mice were not significantly different from controls. There were no statistically significant differences between control and PLE groups on any maternal or litter measure (He et al. 2003; data not shown). Figure 2B shows that control, and low-, and moderate-dose PLE produced significant concentration-dependent increases in [BPb] from PN7–21 with peak [BPb] of ≤ 1, 10, and 26 μg/dL, respectively. By PN60, the [BPb] in PLE mice were not significantly different from controls.

### Body weights

**Figure 3:**
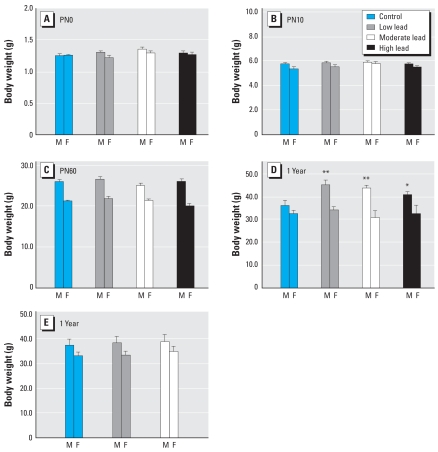
Body weights of GLE (*A–D*) and PLE (*E*) offspring from birth to 12 months of age. There were no significant treatment-related differences in body weight for GLE males (M) or females (F) at (*A*) birth, (*B*) PN10, and (*C*) PN60. However, by 12 months of age (*D*) GLE males but not females were significantly heavier than controls. Low-dose GLE males were significantly heavier than high-dose GLE males. (*E*) There were no significant treatment-related differences in body weight for PLE mice during development, aging, (data not shown) or at 12 months of age. Values are mean ± SE. *p < 0.05; **p < 0.01.

Figure 3 shows that GLE had no significant effect on body weight at PN0, 10, and 60, although by PN60, males in all groups weighed significantly more than females (Figure 3C). Similarly, there were no significant treatment-related differences in body weight at 6 or 10 months of age (data not shown) or at 1 year in female GLE mice (Figure 3D). In contrast, year-old low- (+26%), moderate-(+21%), and high-dose (+13%) male GLE mice weighed significantly more than controls (Figure 3D). There were no significant treatment-related effects of PLE on body weight during development, aging, or at 12 months of age (Figure 3E).

### Exploratory activity and running wheel activity

**Figure 4:**
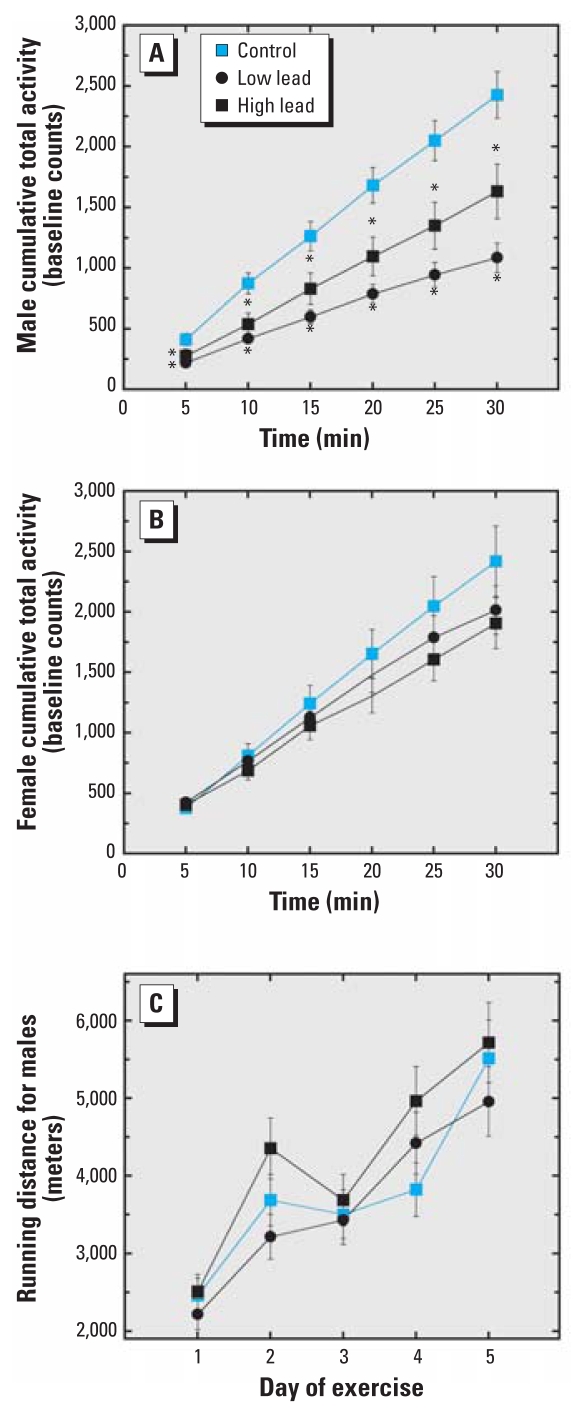
Spontaneous locomotor activity and exercise wheel activity in year-old GLE mice. (*A*) Compared with controls, both low-dose and high-dose GLE males were significantly less active. Except at 5 and 10 min, the low-GLE mice were significantly slower than high-dose GLE mice. (*B*) There were no significant differences between groups in female mice. (*C*) There were no significant differences between groups of male mice in their spontaneous wheel running activity. Values are mean ± SE. *p < 0.05.

When placed in an activity chamber for 30 min for the first time, 1-year-old control male (Figure 4A) and female mice (Figure 4B) exhibited similar levels of exploratory activity. However, male mice in the low- and high-dose GLE groups were significantly less active than control mice: total mean decrease was 52% and 35%, respectively (Figure 4A). Low-dose GLE males were significantly less active than high-dose GLE males. In contrast, there were no treatment-related differences for female mice (Figure 4B). Because year-old GLE males weighed more than age-matched control males (Figure 3D), we addressed the possibility that excess body weight made the GLE males lethargic and less active. This hypothesis was not confirmed, as there were no treatment-related differences in male running wheel activity (Figure 4C).

### Amphetamine-induced motor activity

**Figure 5:**
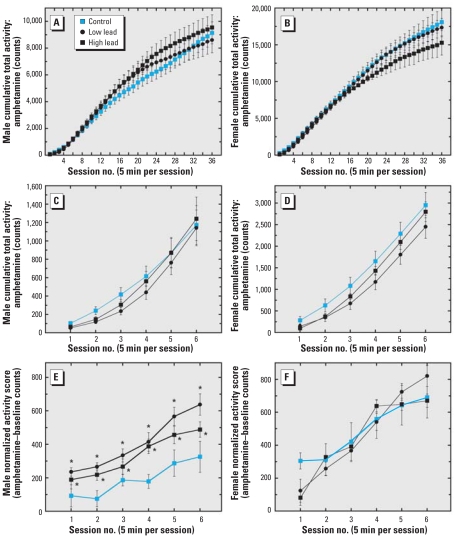
Amphetamine-induced motor activity in year-old GLE mice. There were no apparent differences between treatment groups for either male (*A*) or female (*B*) mice, although males exhibited significantly less cumulative amphetamine-induced activity (3 mg/kg, ip) than females over the 3-hr period. There were no apparent differences in amphetamine-induced motor activity between treatment groups for male (*C*) or female mice (*D*), although males had significantly less cumulative activity than females during the first 30 min. The amphetamine-minus-baseline activity score for the first 30 min revealed that GLE males exhibited a significantly more rapid onset and greater response to amphetamine relative to age-matched controls (*E*), whereas the females showed no overall change in the net amphetamine behavioral response (*F*). Values are mean ± SE. *p < 0.05.

Amphetamine-induced motor activity was recorded in year-old mice (Figure 5). Initial data analysis suggested that there were no differences between treatment groups, although males (Figure 5A, C) were 50% less active after amphetamine challenge than females (Figure 5B, D). Because the baseline exploratory activity in GLE males (Figure 4A) but not in females (Figure 4B) was significantly decreased, we conducted a more detailed analysis of the first 30 min of amphetamine-induced motor activity. Figure 5C and D reveals no apparent between-group differences in amphetamine-induced motor activity. However, when we subtracted the baseline activity counts from the amphetamine-induced activity counts for the first 30 min, the net motor activity revealed that low- and high-dose GLE males exhibited a significant (2-fold) overall heightened sensitivity to amphetamine stimulation (Figure 5E). GLE females exhibited no overall change in sensitivity to amphetamine-stimulated motor behavior (Figure 5F).

### Rotarod performance

**Figure 6:**
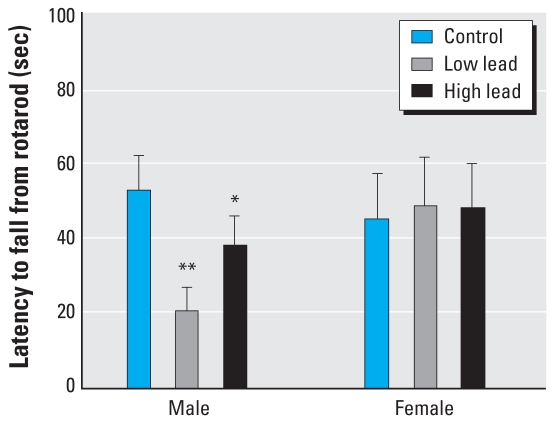
Rotarod performance in year-old GLE mice. Control male and female mice performed similarly on the rotarod task. Compared with age-matched controls, GLE males had significantly shorter latencies to fall from the rotarod. Low-dose GLE males fell off the rotarod significantly faster than high-dose GLE males. In contrast, there were no treatment-related differences between female mice. Values are mean ± SE. *p < 0.05; **p < 0.01.

The mean latency to fall from the rotarod was not different in year-old male and female control mice or female GLE mice (Figure 6). In contrast, all GLE males had significantly shorter latencies to fall from the rotarod compared with age-matched controls. In addition, the mean rotarod performance of low-dose GLE males was significantly poorer than that of high-dose GLE males.

### Striatal and forebrain DA metabolism

**Figure 7:**
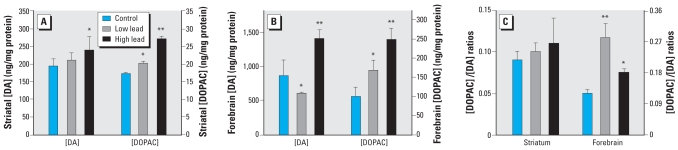
Striatal and forebrain content of DA and DOPAC and DA utilization in year-old GLE male mice. (*A*) High-dose GLE increased striatal [DA] and both GLE doses elevated striatal [DOPAC]. (*B*) Low-GLE decreased forebrain [DA], whereas high-dose GLE increased forebrain [DA]. Both low and high-dose GLE increased forebrain [DOPAC]. (*C*) Dopamine utilization ([DOPAC]/[DA]) ratio was unchanged in the striatum of GLE mice, whereas the ratio increased in the fore-brain of both low- and high-dose GLE mice. Values are mean ± SE. *p < 0.05; **p < 0.01.

High-dose GLE significantly increased striatal [DA] by 23% and [DOPAC] by 58%, whereas low-dose GLE significantly elevated [DOPAC] by 17% (Figure 7A). Striatal DA utilization was unchanged in GLE mice (Figure 7C). In the forebrain, high-dose GLE significantly increased [DA] by 63% and [DOPAC] by 149%, and low-GLE significantly increased [DOPAC] by 69% (Figure 7B). However, in the forebrain low-GLE decreased [DA] by 30%, and both low- and high-dose GLE increased DA utilization (Figure 7C). Moreover, the increased forebrain DA utilization was significantly greater in low-dose (+133%) than in high-dose (+50%) GLE mice.

## Discussion

We report six novel results. First, a new and toxicologically relevant murine model of human equivalent low-level GLE was established. Second, the long-term physiological, behavioral, and neurochemical effects of low-level GLE were examined in year-old male and female mice. Third, sex-specific increases in body weight were observed in year-old GLE male mice. Fourth, male-specific alterations in spontaneous and amphetamine-induced motor behaviors were found in year-old GLE mice. Fifth, alterations in striatal and forebrain DA metabolism were present in year-old male GLE male mice. Sixth, and most important, GLE produced nonmonotonic dose-dependent responses because the alterations were consistently larger in the low-dose than in the high-dose GLE group. These responses are characteristic of inverted U-shaped dose–response curves often observed in lead neuro-toxicity studies (Davis and Svendsgaard 1990).

One of the most compelling findings in the present study is that GLE acted as a delayed obesogen. Specifically, by 1 year of age male GLE mice were obese (Pizzi and Barnhart 1976), and the weight gain was greater in low-and moderate-dose than in high-dose GLE mice. This nonmonotonic characteristic is supported by reports showing that lifetime exposure to 5- to 20-fold higher lead levels either did not affect body weight of 14-month-old male or female rats (Verlangieri 1979) or decreased it in adult rats (Carmichael et al. 1981).

Obesity and related disorders such as diabetes and cardiovascular disease have increased dramatically in children and adults during the past 25 years (Hedley et al. 2004). Additionally, epidemiological studies show a strong association between obesity and a variety of cancers (Calle and Kaaks 2004). Although the molecular mechanism responsible for the lead-induced delayed obesity is unknown, there are several candidates. First, genetic polymorphisms in the vitamin D receptor, insulin-induced gene 2 (*INSIG2*), and FTO genes (fat mass and obesity associated genes) (Frayling et al. 2007; Ye et al. 2001) have been linked to obesity and metabolic disorders. The possibility that GLE produced polymorphisms in the vitamin D receptor is suggested by findings that lead inhibits vitamin D_3_ receptor–regulated calcium metabolism (Schanne et al. 1992), and vitamin D receptor polymorphisms elevate [BPb] and tibial [BPb] (Rezende et al. 2008; Schwartz et al. 2000). Second, hypothalamic dopaminergic receptors are associated with genetic obesity in mice (el-Refai and Chan 1986). Developmental studies have linked lead to hypothalamic dopaminergic dysfunction and altered hypothalamic–pituitary–adrenal axis (Cory-Slechta et al. 2004; Govoni et al. 1984). Third, the environmental obesogen hypothesis states that dietary and environmental chemicals disrupt endocrine signaling pathways and thereby contribute to obesity (Baillie-Hamilton 2002; Heindel 2003). Recent studies suggest that fetal exposure to organotins produce obesity in male rats by activating certain nuclear receptors (Grun et al. 2006). Thus, lead-induced delayed obesity could occur via a variety of endocrine/ metabolic mechanisms.

The spontaneous locomotor activity and rotarod performance were decreased in GLE male mice, with greater effects seen in the low-dose than in the high-dose GLE group. These curvilinear results are consistent with “negative” dose–response curves, as high-dose GLE produced less deviation from control than did low-dose GLE (Davis and Svendsgaard 1990). Because year-old GLE males were significantly heavier than controls, we tested whether these mice were lethargic and less active on a running wheel. Because all three groups ran comparable distances, the results indicate that the GLE-induced motor alterations were not caused by a lack of motivation or inefficient locomotion.

We hypothesized that the novel environment differentially increased the stress level of aged GLE male mice, which subsequently altered dopaminergic signaling and decreased locomotor activity. Consistent with this proposal are recent data showing that moderate-to-high-level lead exposure during gestation and throughout lactation produced *a*) elevated basal corticosterone concentrations in male offspring, *b*) a decrease in novel exploratory behavior of males, and *c*) decreased fixed-interval response rates in males (Cory-Slechta et al. 2004; Moreira et al. 2001; Virgolini et al. 2004). However, the role of DA is still unknown, as both adult male and female rats exhibited dopaminergic dysfunction in several brain regions (Cory-Slechta et al. 2004). In summary, our findings reveal that low-level GLE produced the most profound and enduring alterations in locomotor activity, another example of nonmonotonic responses. Furthermore, the results suggest that a coordinated dysregulation of the hypothalamic–pituitary–adrenal axis and dopaminergic systems might underlie these changes.

Amphetamine enhances low rates of responding and depresses high rates of responding, which produces the classic inverted U-shaped dose–response effect (Glick and Milloy 1973). Because year-old GLE male but not female mice had decreased locomotor activity compared with age- and sex-matched controls, we reasoned that amphetamine would differentially increase locomotor behavior in GLE males and produce no differential locomotor effect in year-old female mice. Indeed, amphetamine increased the locomotor activity of both low- and high-dose GLE mice compared with controls; larger effects occurred in low-dose GLE mice. Although the DA transporter is a major target site of amphetamine (Madras et al. 2005), dopamine D_3_ receptors in the ventral striatum also regulate the sensitivity to the locomotor-stimulating effects of amphetamine (McNamara et al. 2006). Although alterations in striatal DA metabolism as well as D_1_ and D_2_ dopamine receptors occur in developmentally lead-exposed rats (Cory-Slechta 1997; Ma et al. 1999), there are at present no studies on the dopamine D_3_ receptors.

Developmental lead exposure is associated with attention-deficit/hyperactivity disorder (ADHD) in children (Bellinger et al. 1994; Braun et al. 2006), which is among the most common childhood and adult neurological disorders (Kessler et al. 2006). Neuroanatomical and functional abnormalities in the prefrontal cortex are reported to underlie this deficit (Barkley et al. 1992). Moreover, recent functional magnetic resonance imaging studies revealed a diminished activation in the frontal cortex of young adults after developmental lead exposure (Yuan et al. 2006). It is unknown whether GLE and/or early PLE produce the greatest risk for childhood ADHD. The contribution of GLE to adult-onset ADHD, which also has a 1.6 male–female odds ratio as childhood ADHD (Kessler et al. 2006), is unknown. Our amphetamine-stimulated locomotor results in year-old male GLE mice are consistent with the U-shaped therapeutic outcome for ADHD and suggest that low-level GLE merits consideration as a causative factor to lead-induced ADHD in children and adults.

Both groups of aged GLE male mice were impaired on the rotarod, which is a test of interlimb balance and coordination. Low-dose GLE males were significantly more impaired than high-dose GLE males. Consistent with the negative dose–response curve, rotarod activity was unchanged in adult male rats exposed to 5- to 40-fold higher lead levels during gestation and lactation or lifetime (Ma et al. 1999; Moreira et al. 2001). These rotarod results are reminiscent of the poorer fine motor control, visual motor function, and postural balance found in children, adolescents, and young adults with low-to-moderate developmental lead exposure (Baghurst et al. 1995; Bhattacharya et al. 1990, 2006; Ris et al. 2004; Wasserman et al. 2000; Winneke et al. 1990). Interestingly, there is an increased risk of neuromotor deficits in males (Ris et al. 2004). Our results suggest that low-level GLE contributes to persistent neuromotor and balance deficits and is a risk factor for injuries in older males.

Because body weight and behavioral differences were found only in year-old GLE male mice, neurochemical analyses were conducted in these mice. GLE female mice are being aged for future studies. Numerous studies have reported changes in adult rat brain DA metabolism after moderate-to-high post-natal or lifetime lead exposure (Cory-Slechta 1997; Ma et al. 1999). However, the present report is the first study to use a GLE model and examine neurochemical changes in aged mice. We found that low-dose GLE decreased forebrain [DA] and increased striatal and forebrain [DOPAC], whereas high-dose GLE increased both striatal and forebrain [DA] and [DOPAC]. These GLE-induced changes resulted in nonmonotonic increases in fore-brain DA utilization but no change in striatal DA utilization. The inverted U-shaped dose-response function for forebrain DA utilization correlates with the GLE-induced alterations in weight gain and neuromotor functions. For two reasons it is unlikely that these dopaminergic changes resulted from a direct effect of lead in year-old mice. The peak brain [Pb] in the low- and high-dose GLE mice on PN10 were equivalent to 0.44 and 1.2 μM, respectively, and the brain [Pb] is not different from controls after PN30 (data not shown). In addition, 3–250 μM Pb^2+^ is required to inhibit rat brain tyrosine hydroxylase activity, depolarization-evoked and spontaneous DA release, and DA uptake (Jadhav and Ramesh 1997; Minnema et al. 1986; Ramsay et al. 1980). Thus, further multidisciplinary molecular, biochemical, bioinformatic, and imaging studies are needed to support our findings and provide mechanistic insight.

In summary, our results show that GLE mice with peak [BPb] ≤ 10 μg/dL, the current low level of concern (CDC 1991), have permanent sex-specific motor abnormalities and late-onset obesity. The nonmonotonic dose-dependent responses reveal that low-level GLE produces the most adverse effects. These data raise complex issues for risk assessment and indicate that lifetime measures of dose–response toxicant exposure should be a component of the neurotoxic risk assessment process.

## Figures and Tables

**Table 1:** Maternal and litter measures for control C57BL/6 mice.^a^

Maternal and litter measures	Outcome results
Maternal	
Dams’ fluid consumption for 14 days prior to mating (mL/day)	4.78 ± 0.24
Dams’ fluid consumption during gestation (mL/day)	5.32 ± 0.32
Dams’ fluid consumption from PN1 until PN10 (mL/day)	6.81 ± 0.53
Dams’ weight 14 days prior to mating (g)	19.91 ± 0.53
Dams’ weight at mating (g)	21.12 ± 0.39
Dams’ weight gain during pregnancy (g)	6.23 ± 0.49
Mating success rate (%)	91.6 ± 1.7
Litter	
Length of gestation (days)	19.5 ± 0.2 days
Mean litter size (pups)	9.8 ± 0.5 pups
Sex distribution at birth	54% males; 46% females
Litter mortality (dead pups/litter)	0.6 ± 0.2
Pup mortality during lactation (%)	9.4 ± 0.3

a Mean ± SE values are from 12 to 17 control dams and litters.
